# Structural insights into ligand recognition, activation, and signaling of the α_2A_ adrenergic receptor

**DOI:** 10.1126/sciadv.abj5347

**Published:** 2022-03-04

**Authors:** Jun Xu, Sheng Cao, Harald Hübner, Dorothée Weikert, Geng Chen, Qiuyuan Lu, Daopeng Yuan, Peter Gmeiner, Zheng Liu, Yang Du

**Affiliations:** 1 Kobilka Institute of Innovative Drug Discovery, School of Life and Health Sciences, Chinese University of Hong Kong, Shenzhen, 518172, China.; 2 Department of Chemistry and Pharmacy, Medicinal Chemistry, Friedrich-Alexander University, Nikolaus-Fiebiger-Straße 10, 91058 Erlangen, Germany.; 3 Beijing Advanced Innovation Center for Structural Biology, School of Medicine, Tsinghua University, Beijing 100084, China.

## Abstract

The α_2A_ adrenergic receptor (α_2A_AR) is a G protein (heterotrimeric guanine nucleotide–binding protein)–coupled receptor that mediates important physiological functions in response to the endogenous neurotransmitters norepinephrine and epinephrine, as well as numerous chemically distinct drugs. However, the molecular mechanisms of drug actions remain poorly understood. Here, we report the cryo–electron microscopy structures of the human α_2A_AR-GoA complex bound to norepinephrine and three imidazoline derivatives (brimonidine, dexmedetomidine, and oxymetazoline). Together with mutagenesis and functional data, these structures provide important insights into the molecular basis of ligand recognition, activation, and signaling at the α_2A_AR. Further structural analyses uncover different molecular determinants between α_2A_AR and βARs for recognition of norepinephrine and key regions that determine the G protein coupling selectivity. Overall, our studies provide a framework for understanding the signal transduction of the adrenergic system at the atomic level, which will facilitate rational structure-based discovery of safer and more effective medications for α_2A_AR.

## Teaser

Active α_2A_ adrenergic receptor structures revealed distinct agonist activation and facilitate design of therapeutics for α_2A_AR.

## INTRODUCTION

The adrenergic receptors (adrenoceptors) are a class of G protein (heterotrimeric guanine nucleotide–binding protein)–coupled receptors (GPCRs) that mediate the physiological actions of the endogenous catecholamines norepinephrine and epinephrine (*1*, *2*). There are nine distinct adrenoceptors in mammalian species, which are grouped into three main classes on the basis of their amino acid sequences and biological properties: α_1_ (α_1A_, α_1B_, and α_1D_), α_2_ (α_2A_, α_2B_, and α_2C_), and β (β_1_, β_2_, β_3_) adrenoceptors (*2*, *3*). The α_2_ adrenoceptors modulate a wide range of physiological functions, including the heart rate, blood pressure, regulation of blood glucose, insulin homeostasis, and analgesia, and they are implicated in mediating the presynaptic feedback inhibition of neurotransmitter release from noradrenergic nerve terminals (*4*–*6*).

All three α_2_ subtypes are traditionally known to couple primarily to the Gi/o protein family and inhibit the activity of adenylyl cyclase, resulting in a decrease in intracellular cyclic adenosine monophosphate (cAMP) levels (*4*, *7*). However, there is evidence suggesting that α adrenoceptors can also functionally couple to Gs (*7*–*10*). In addition to G proteins, α_2_ adrenoceptors are also subject to agonist-dependent phosphorylation by G protein-coupled receptor kinases (GRKs), followed by the coupling of β-arrestins, which, in turn, leads to receptor internalization and G protein–independent signaling (*11*–*13*). Recent pharmacological studies have characterized a number of biased agonists that preferentially activate one of the signaling pathways in favor of the others for several GPCRs, including the μ-opioid receptor, the angiotensin II receptor type 1, and the dopamine D_2_ receptor (*14*). Since activation of multiple signaling pathways may lead to functional profiles conferring both beneficial and adverse effects, biased agonists may markedly reduce side effects (*15*). In the past decades, numerous α_2_ agonists have been developed and characterized, and some of them have, for example, been used in anesthesia, in pain management, and for the treatment of hypertension (*16*, *17*). Functional and pharmacological studies show that structurally different α_2_ agonists (e.g., catecholamines, imidazolines, and azepines) display distinct efficacies in the activation of G proteins (*18*, *19*), while the signaling behavior of these α_2_ agonists toward the β-arrestin pathway remains poorly understood.

In addition to downstream signaling diversity, both α_2_ and β adrenoceptors respond to norepinephrine and epinephrine. While the molecular mechanisms of drug action (including norepinephrine and epinephrine) on β adrenoceptors (β_1_AR and β_2_AR) have been extensively investigated (*20*–*25*), relatively little is known about the structural basis of drug action and activation mechanism of the α_2_ adrenoceptors, with the only reported structures being the inactive α_2A_ adrenergic receptor (α_2A_AR) and α_2C_AR and the active α_2B_AR–G protein complex structures (*9*, *26*, *27*). Here, we report the high-resolution cryo–electron microscopy (cryo-EM) structures of the α_2A_AR-GoA complex bound to four chemically different agonists. In combination with structure-guided mutagenesis and functional analysis, our studies provide a basis for understanding the molecular mechanisms of ligand recognition, activation, and signaling of the α_2A_AR using pathway-specific assays.

## RESULTS

### Cryo-EM structures of the α_2A_AR-GoA complex bound to norepinephrine and imidazoline agonists

**Fig. 1.**
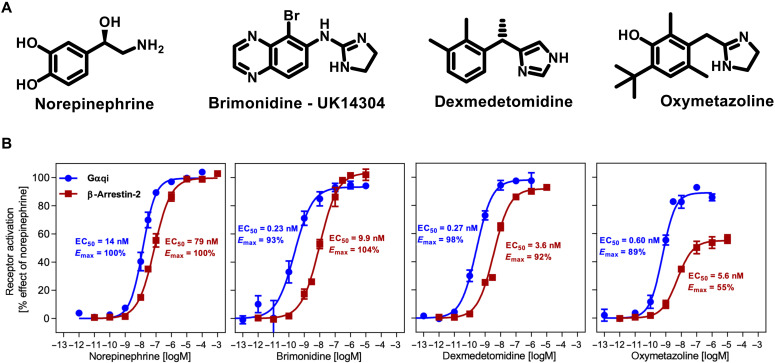
Structure and function of α_2A_AR agonists. (**A**) Chemical structures of norepinephrine (Norepi), brimonidine (BRI) or UK14304, dexmedetomidine (DEX), and oxymetazoline (OXY). (**B**) Concentration-response curves of different agonists toward G protein activation (blue curve) and β-arrestin-2 recruitment (red curve) measured by a cell-based Gqi-inositol phosphate accumulation assay and the PathHunter assay respectively. Data are presented as means ± SEM of 4 to 10 independent experiments with repeats in duplicate.

In this study, we determined the structure of the α_2A_AR-GoA complex bound to four chemically different agonists including the endogenous agonist norepinephrine and three imidazoline derivatives brimonidine, dexmedetomidine, and oxymetazoline (Fig. 1A; denoted as Norepi, BRI, DEX, and OXY, respectively). Both BRI and DEX are highly selective synthetic α_2A_AR agonists over the α_1A_AR subtype, but they have different therapeutic applications (*16*). BRI is commonly used to treat ocular hypertension and open-angle glaucoma, while DEX is an anxiety-reducing and pain-relieving drug, and most notable for its clinical use for providing sedation without compromising the airway and depressing respiration (*16*). OXY is generally available as a nasal decongestant that acts on both α_2A_AR and α_1A_AR. Previous functional and pharmacological studies of these α_2A_AR agonists have been largely restricted to the G protein–dependent signaling pathway, while very little is known regarding their signaling profiles in the β-arrestin pathway. Thus, we performed β-arrestin recruitment assays and compared the results to G protein activation by α_2A_AR in a well-established downstream signaling assay (IP-one) using a chimeric Gqi protein and Norepi as a reference agent (Fig. 1B and table S2). Our results show that all drugs display full agonist activity in the Gqi-mediated signaling, except for the slightly lower efficacy for OXY (89% of Norepi) (Fig. 1B). Of interest, OXY also shows only partial agonist properties of arrestin recruitment (55% *E*_max_ of Norepi), which is in contrast to the full agonist properties for the other three drugs, suggesting that OXY is a potential Gi/o-biased agonist at the α_2A_AR.

**Fig. 2.**
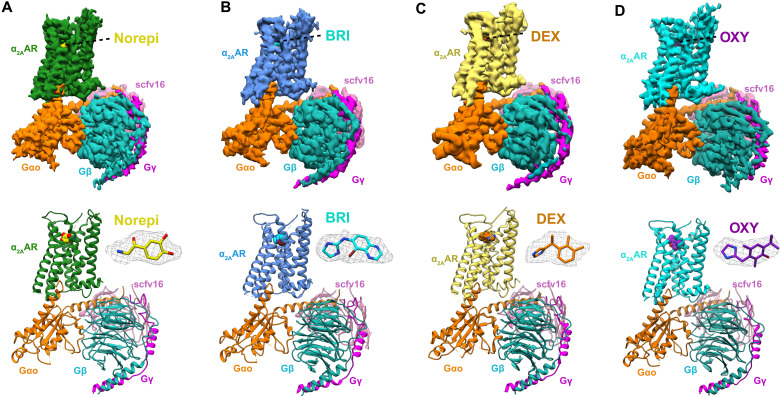
Cryo-EM structures of Norepi, BRI-, DEX-, and OXY-bound α_2A_AR-GoA complexes. Cryo-EM density maps and models of the α_2A_AR-GoA complex bound to Norepi (**A**), BRI (**B**), DEX (**C**), and OXY (**D**). The densities of the agonists (shown as sticks) are depicted as gray meshes. The maps are colored according to different subunits.

To gain insights into receptor recognition by different ligands, we assembled the complex of human α_2A_AR and the heterotrimeric GoA in the presence of the four different agonists. We kept the long intracellular loop 3 (ICL3) and used the wild-type receptor for our structural studies (fig. S1A). The complexes were prepared as described in Materials and Methods, and the agonist-bound α_2A_AR formed a stable nucleotide-free complex with GoA in the presence of an antibody fragment scFv16 (fig. S1B) (*28*). We obtained the cryo-EM maps of α_2A_AR-GoA-scFv16 in complex with the four different agonists with overall resolutions of 3.2 Å (Norepi), 3.0 Å (BRI), 3.6 Å (DEX), and 3.4 Å (OXY) (Fig. 2, figs. S2 and S3, and table S1). These maps allowed us to build the model of the transmembrane domain of the α_2A_AR, the GoA heterotrimer, and the scFv16. The maps also showed well-defined densities for each of the agonists in the orthosteric pocket (Fig. 2 and fig. S3). We did not observe clear densities for the first ~30 amino acids and the ICL3 of α_2A_AR, suggesting disordered conformations of these regions. The α-helical domain of Gαo was also poorly resolved because of its flexibility as observed in most cryo-EM structures of GPCR–G protein complexes (*29*, *30*).

### Orthosteric binding pocket of α_2A_AR

**Fig. 3.**
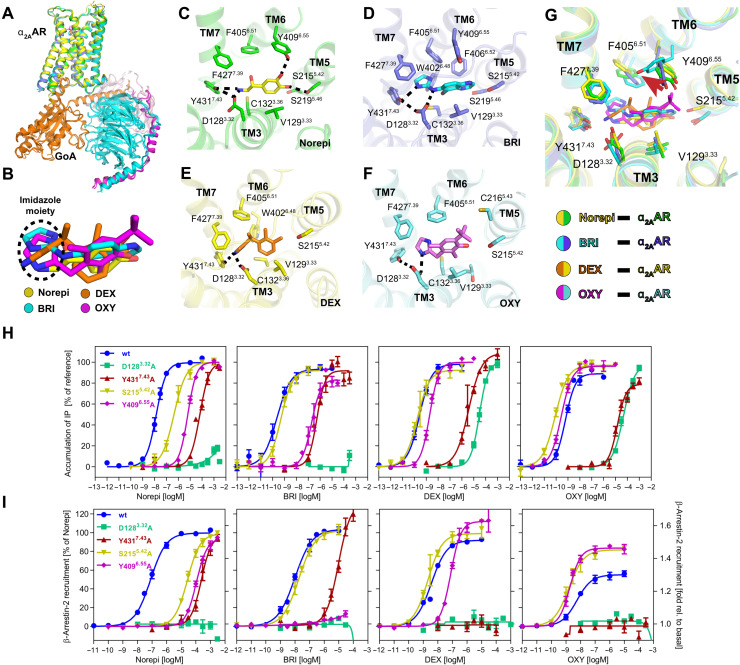
Orthosteric binding pocket of active α_2A_AR bound to different agonists. (**A**) Alignment of four structures of α_2A_AR signaling complexes. (**B**) Superposition of Norepi, BRI, DEX, and OXY from cryo-EM structures. The imidazole moiety is highlighted by the dashed circle. (**C** to **F**) Detailed interactions of Norepi (C), BRI (D), DEX (E), and OXY (F) with α_2A_AR. Residues within 4 Å of agonist are shown in sticks. The polar interactions are indicated by black dashed lines. (**G**) Superposition of the α_2A_AR orthosteric binding pocket residues bound to four different agonists. (**H** and **I**) Concentration-response curves of different agonists for G protein activation (H) and β-arrestin-2 recruitment (I) for wild-type (wt) α_2A_AR and receptor mutants D128^3.32^A, Y431^7.43^A, S215^5.42^A, and Y409^6.55^A, respectively. Except for the activation of D128^3.32^A, which is normalized to DEX for G protein activation and relative to basal for arrestin recruitment, receptor activation is shown relative to the maximum effect of Norepi. Data are presented as means ± SEM of 3 to 11 independent experiments with repeats in duplicate.

Despite the chemical diversity of the agonists, the α_2A_AR-GoA complexes bound to Norepi or the imidazoline agonists have very similar overall structures (Fig. 3A), and all four drugs occupy a similar pose in the orthosteric pocket (Fig. 3B). However, there are substantial differences for their interactions with surrounding residues. Figure 3 (C to G) shows detailed interactions of each agonist with the residues located in the α_2A_AR orthosteric pocket, which consists of the extracellular parts of TM3, TM5, TM6, and TM7.

Norepi binds to α_2A_AR primarily through two polar interaction networks. First, the amino-ethanol group of Norepi interacts with D128^3.32^ and Y431^7.43^ (superscript indicates Ballesteros-Weinstein numbering) via a salt bridge and a hydrogen bond (Fig. 3C). This polar interaction network with TM3 and TM7 is also observed for all three imidazoline agonists (Fig. 3, D to F). Consistently, mutations D128^3.32^A and Y431^7.43^A lead to a notable loss of activity for all drugs in both G protein signaling and arrestin recruitment (Fig. 3, H and I, and table S2). D^3.32^ is highly conserved in aminergic GPCRs, and structural and biochemical studies showed that it also plays pivotal roles in ligand recognition and activation of dopamine receptors and β adrenergic receptors (*25*, *31*, *32*). Notably, OXY can only form a direct interaction with D128^3.32^ but not Y431^7.43^ (Fig. 3F), resulting in a relatively weaker interaction with TM7. This may account for its partial agonism in arrestin recruitment (Figs. 1B and 3, H and I). Alanine replacement of Y431^7.43^ completely abolishes the arrestin recruitment induced by OXY and DEX, while it preserves G protein signaling at high ligand concentration, suggesting a potential role of Y431^7.43^ in mediating pathway-specific signaling bias of α_2A_AR (Fig. 3, H and I, and table S2).

Second, the para- and meta-phenolic hydroxyls of the Norepi phenyl ring form hydrogen bonds with S215^5.42^ and Y409^6.55^, respectively, which is in accordance with the recent docking pose (*9*). Consistently, mutation of S215^5.42^A and Y409^6.55^A markedly reduced the activity of Norepi (Fig. 3, H and I, and table S2). In contrast, BRI is not able to form stable hydrogen bonds with either S215^5.42^ or Y409^6.55^, in spite of the polar nitrogen atoms of the bicyclic aromatic moiety (Fig. 3D). Indeed, mutation S215^5.42^A has little effect on the activity of BRI. However, Y409^6.55^A reduces the EC_50_ by ~800-fold on Gqi signaling and completely abolished the arrestin recruitment induced by BRI (Fig. 3, H and I, and table S2). This is likely due to breakage of the π-π interactions between Y409^6.55^ and the bicyclic aromatic moiety of BRI. It is also possible that there is a water-mediated hydrogen bond between BRI and Y409^6.55^. Besides, the greater reduction of activity in arrestin recruitment than G protein activation for Norepi and BRI suggests that Y409^6.55^ also plays a potential role in regulating pathway-specific signaling of α_2A_AR.

The phenyl ring of DEX lacks the two hydroxyls of catecholamines; instead, only two methyl groups and no polar substituents are present. As a result, DEX interacts with TM5 and TM6 mainly through hydrophobic interactions (Fig. 3E), which is similar to what was observed in the α_2B_AR (*27*). Mutagenesis data show that S215^5.42^A has no influence for the potency of DEX, and Y409^6.55^A reduces its potency less compared with Norepi and BRI (Fig. 3, H and I, and table S2). Although OXY comprises a phenolic hydroxyl, it cannot form a stable hydrogen bond with S215^5.42^ (Fig. 3F). The Y409^6.55^ side chain displays a unique conformation in the OXY-bound structure by rotating around 70° toward TM7, likely due to the presence of the tert-butyl substituent of OXY (Fig. 3G). This unique bulky hydrophobic group may contribute to the partial agonist properties for arrestin recruitment of OXY because of the incompatibility with the hydrophilic properties of Y409^6.55^ and S215^5.42^. Indeed, both S215^5.42^A and Y409^6.55^A mutations lead to a slightly increased activity of OXY, in particular, the efficacy in arrestin recruitment is markedly enhanced, which is different from what is observed for other agonists (Fig. 3, H and I, and table S2). The agonist-dependent functional impacts of these mutations, particularly for Y409^6.55^, suggest important but complicated roles of these residues in modulating biased signaling of α_2A_AR. Notably, recent structural, functional, and computational studies also suggested that the residue in position 6.55 plays an important role in regulating bias for the β_2_AR (*24*, *33*). It should be noted that the functional effects of these mutations on the α_2A_AR are not due to changes in receptor expression, since receptor mutants S215^5.42^A, Y409^6.55^A, and D128^3.32^A show surface expression comparable to wild type and expression is only slightly reduced for Y431^7.43^ (fig. S4).

### Recognition of Norepi by α_2A_AR and β adrenoceptors

**Fig. 4.**
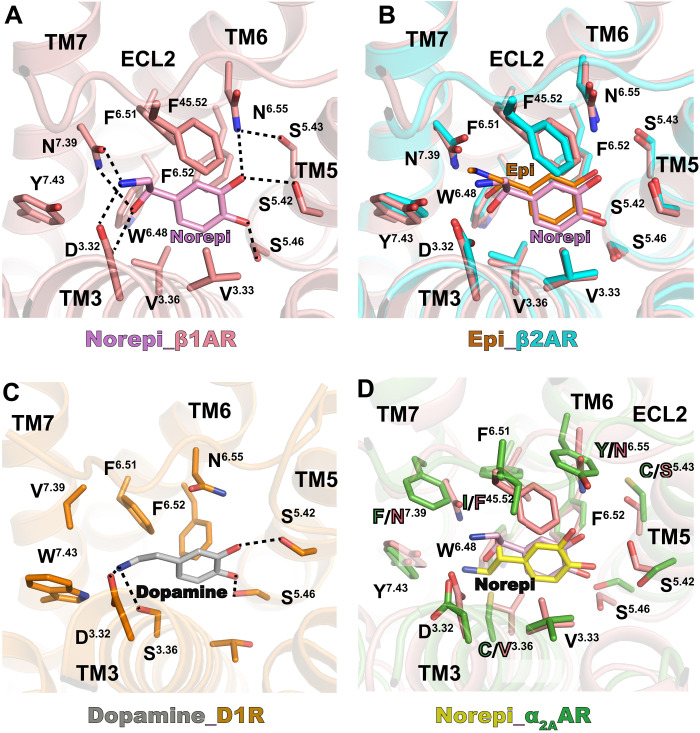
Comparison of Norepi binding for α_2A_ and β adrenergic receptors. (**A**) Detailed interactions of Norepi with β_1_AR [Protein Data Bank (PDB) code: 7BU6]. Residues within 4 Å of agonist are shown in sticks. The polar interactions are indicated by black dashed lines. (**B**) Superposition of orthosteric pockets of β_1_AR-Norepi and β_2_AR-Epi (epinephrine) (PDB code: 4LDO). (**C**) Detailed interactions of dopamine with D1R (PDB code: 7CKZ). The polar interactions are indicated by black dashed lines. (**D**) Superposition of orthosteric pockets of β_1_AR-Norepi and α_2A_AR-Norepi. Residues within 4 Å of agonist are shown in sticks.

Different levels of Norepi in human body could result in distinct physiological behaviors because of its highest affinity for α_2_ adrenoceptors and lowest affinity for β adrenoceptors (*5*, *6*). It is therefore of interest to understand the molecular basis of Norepi recognition by the two adrenergic subfamilies that have opposite functional effects. Comparison of Norepi-bound β_1_AR and epinephrine-bound β_2_AR shows identical binding pockets (Fig. 4, A and B). Norepi interacts with β adrenoceptors mainly through two polar networks, similarly as observed in α_2A_AR (Figs. 3C and 4A). The hydrogen bonds with the conserved D^3.32^ and S^5.42^ are maintained in β adrenoceptors, while the para-hydroxyl of Norepi forms additional hydrogen bond with S^5.46^. This D^3.32^-S^5.42^-S^5.46^ motif is also found to be involved in polar interactions between dopamine and the D1 dopamine receptor (Fig. 4C) (*32*). Although S^5.46^ is not involved in the hydrogen-bonding interaction with Norepi in α_2A_AR, previous mutagenesis data showed that S^5.46^A markedly reduced the potency of epinephrine to inhibit forskolin-stimulated cAMP accumulation (*4*), suggesting a conserved role of the D^3.32^-S^5.42^-S^5.46^ motif also in the α_2A_AR.

On the other hand, the S^5.43^ in β adrenoceptors, which is a cysteine in α_2A_AR, participates in the polar network through hydrogen bonding with N^6.55^ (Fig, 4, A and B). The two aromatic residues in TM6 (Y^6.55^) and TM7 (F^7.39^) of α_2A_AR are also replaced with two asparagine residues (N^6.55^ and N^7.39^) in β adrenoceptors, both of which are involved in the polar interaction networks with Norepi (Fig. 4, A and B). Simulation studies showed that the direct interaction of the amino-ethanol of Norepi and other β adrenergic compounds with N^7.39^ was very prominent (*33*), while this polar interaction with residue 7.39 almost gets lost in α_2A_AR because of the sequence difference (Fig. 4D). By contrast, the amino-ethanol of Norepi forms direct interaction with Y^7.43^ in α_2A_AR (Fig. 3C), which is probably an analog to the polar interaction with N^7.39^ in β adrenoceptors. Notably, the two aromatic residues (Y^6.55^ and F^7.39^) in α_2A_AR, together with F^6.51^, F^6.52^, Y^7.43^, and W^6.48^, form an aromatic cage, which may play key roles in high-affinity Norepi binding (Fig. 4D) (*9*). Recent functional studies showed that the F^7.39^N mutation completely abolishes the activity of Norepi, and the Y^6.55^N mutation can substantially reduce the EC_50_ of Norepi for α_2A_AR (*9*). Moreover, the bulkier F^45.52^ in ECL2 of the β adrenoceptors is a smaller isoleucine in the α_2A_AR. Although I ^45.52^ makes weaker hydrophobic contacts with the phenyl ring of norepinephrine than F^45.52^, it may result in different conformational dynamics along the entrance pathway in α_2A_AR, contributing to the higher affinity of Norepi (*22*).

### Activation of the α_2A_AR

**Fig. 5.**
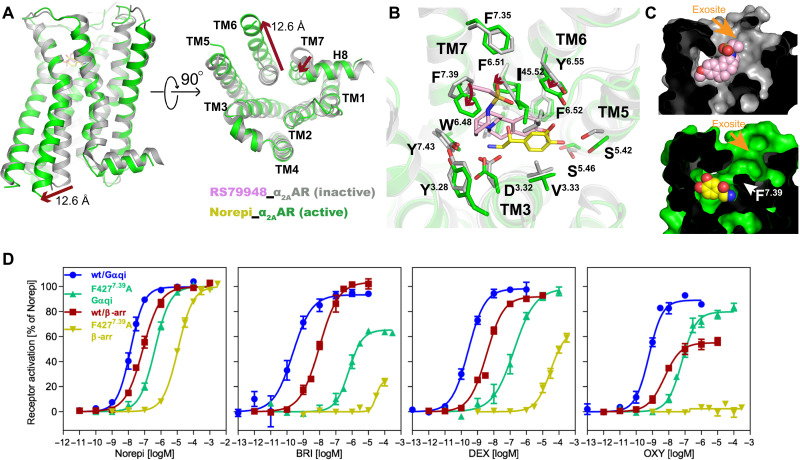
Comparison of inactive and active α_2A_AR. (**A**) Structural comparison of inactive α_2A_AR bound to RS79948 and active α_2A_AR bound to Norepi, with changes highlighted as red arrows. The distance is calculated between positions of Cα of residue 6.29 in TM6. (**B**) Conformational changes within the orthosteric pocket are shown from the extracellular side, with changes highlighted as red arrows. (**C**) Cross sections of α_2A_AR bound to antagonist and agonist are shown, with the interior in black and the exosite highlighted. (**D**) Concentration-response curves of F427^7.39^ mutant for different agonists toward G protein activation and β-arrestin-2 recruitment. Data are presented as means ± SEM of 4 to 10 independent experiments with repeats in duplicate.

Although the first inactive structure of the β adrenoceptor has been determined in 2007 (*34*), the inactive structures of α2 adrenoceptors were solved only recently (*9*, *26*), allowing us to analyze the conformational changes of α_2A_AR from inactive to active states. The outward movement of TM6 (12.6 Å) and inward movement of TM7, which are hallmarks of GPCR activation (*35*, *36*), also occur upon α_2A_AR activation (Fig. 5A). In the orthosteric pocket, the antagonist RS79948 seems to occupy a distinct pose compared with agonists (Fig. 5B). The antagonist RS79948 binds at a further outside position than Norepi and extends to an exosite to form interactions with the extracellular loops (Fig. 5, B and C). The side chains of several residues undergo large rearrangements upon receptor activation, especially for F427^7.39^ (Fig. 5B). The recent α_2A_AR structure bound to the partial agonist RES also shows similar changes in the orthosteric pocket (*9*). F427^7.39^ was proposed to serve as a switching lid of an aromatic cage (together with F^6.51^, F^6.52^, Y^7.43^, and W^6.48^) upon agonist binding (*9*). Indeed, F427^7.39^ displays similar active conformations when bound to different agonists (Fig. 3G), suggesting a pivotal role of the F427^7.39^ conformational change in receptor activation. Likewise, alanine replacement of F427^7.39^ markedly impaired the functions of all agonists, and the imidazoline drugs displayed greater reduction of activities than Norepi (Fig. 5D and table S2). This is consistent with the observation that F427^7.39^ has stronger contacts to the imidazoline ring than the amino-ethanol group of Norepi (Fig. 3G).

Notably, the binding poses for agonists and antagonists are more overlapped for β adrenoceptors (fig. S5A), and most of the interactions with the receptor are identical for agonists and antagonists (*20*). Moreover, the residue at position 7.39 is a smaller asparagine in β adrenoceptors and shows similar side-chain conformations when bound to different ligands (fig. S5A). This asparagine disrupts the aromatic cage for β adrenoceptors (*9*). This disruption seems to allow a few β adrenoceptor agonists with a longer tail, such as salmeterol, to extend to an exosite in the extracellular vestibule, resulting in great subtype selectivity (fig. S5B) (*24*). Whether the extracellular site can be used for novel α_2A_AR agonist design needs further investigation.

In spite of these differences in conformational changes of the orthosteric pocket and in ligand recognition, the α_2A_AR shows similar activation-associated structural changes to the β adrenoceptors in residues that connect the orthosteric pocket to the cytoplasmic surface. The conformational rearrangements of several conserved microswitches, representing the PIF, NPxxY, and DRY motifs, are also similar to that of β adrenoceptors (fig. S5, C to E). These structural features suggest a conserved mechanism for the allosteric coupling between the orthosteric pocket and G protein coupling domain for adrenoceptors.

### G protein coupling interface

**Fig. 6.**
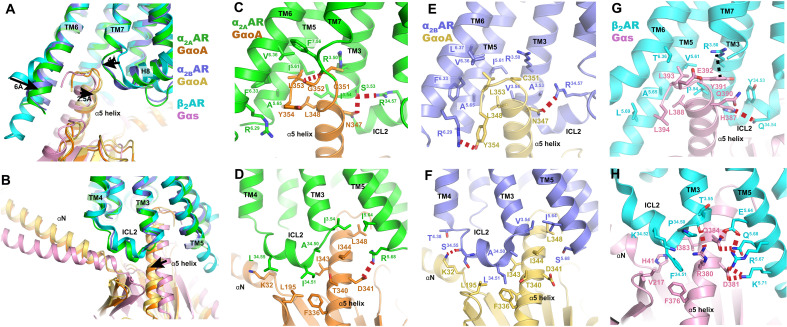
G protein binding interface. (**A** and **B**) Superposition of the G protein coupling interfaces of α_2A_AR-GoA, α_2B_AR-GoA, and β_2_AR-Gs complexes, using receptor for alignment. (**C** to **H**) Detailed interactions of α_2A_AR with Goα (C and D), α_2B_AR with Goα (E and F), and β_2_AR with Gsα (G and H). The polar interactions are indicated by red dashed lines.

Given that α_2_ and β_2_ adrenoceptors have adverse physiological functions, with α_2_ inhibiting the activity of adenylyl cyclase via Gi/o and β_2_AR stimulating it via Gs, they have long served as model systems for studying G protein coupling selectivity. Figure 6 shows the comparison of the coupling interface for GoA with α_2_AR (α_2A_AR and α_2B_AR) and Gs with β_2_AR. Here, we used the Norepi-bound α_2_AR-GoA for comparison with other adrenoceptors, as the interfaces with GoA for different agonist-bound α_2_AR are almost identical (fig. S6A).

The overall interaction interfaces for α_2A_AR-GoA and α_2B_AR-GoA are almost identical (Fig. 6, A and B). The major interaction interface is composed of hydrophobic residues I343^G.H5.15^, I344^G.H5.16^, L348^G.H5.20^, and L353^G.H5.25^ (superscript indicates CGN numbering system) of the Gαo cluster with surrounding hydrophobic residues from TM3, TM5, TM6, and ICL2. However, several differences in polar interactions with the GoA heterotrimer were observed between α_2A_AR and α_2B_AR. For example, the last amino acid Y354^G.H5.26^ of Gαo adopts a distinct side-chain conformation and forms a hydrogen bond with R^6.29^ in α_2B_AR. In contrast, this interaction is not observed in α_2A_AR (Fig. 6, C to F). A hydrogen bond was observed between the backbone carbonyl of F^7.56^ and G352^G.H5.24^ of Gαo. A similar polar interaction was observed between rhodopsin and Gi (*37*), while this interaction pattern is not present in α_2B_AR. In addition, N347^G.H5.19^ of Gαo participates in a hydrogen bond network with S^3.53^ and R^34.57^ in α_2A_AR. However, position 3.53 is an alanine in α_2B_AR and N347^G.H5.19^ of Gαo directly forms a hydrogen bond with R^34.57^. R^5.68^ in α_2A_AR, which is a serine in α_2B_AR, also forms a salt bridge with D341^G.H5.13^ of Gαo. On the other hand, both ICL1 and ICL2 of α_2B_AR form hydrogen bonds with the Gβ subunit and the αN helix via R^12.48^ and S^34.55^, respectively (Fig. 6F and fig. S6B), while these polar interactions were not observed in the α_2A_AR-GoA complex (fig. S6C). These distinct interactions between α_2A_AR-GoA and α_2B_AR-GoA suggest the versatility of Go coupling with GPCRs.

The comparison of α_2_ARs-GoA and β_2_AR-Gs complex structures shows that the relative positions for Gαo and Gαs are different, with the extreme C terminus of Gαo helix α5 shifting 2.5 Å toward TM7 and a large displacement of the αN helix. This is associated with the smaller outward displacement of TM6 and inward movement of TM7 in α_2_ARs, resulting in a smaller cavity formed by TM3/5/6/7 for GoA than for Gs (Fig. 6, A and B). This structural feature is also observed for several other Gi/o-coupled receptors and is believed to be one of the key determinants for the coupling specificity of Gi/o and Gs (*29*, *30*, *37*). However, the orientations of Gi/o proteins coupled to different receptors are also divergent. For example, the extreme C terminus of Gαi helix α5 of M2R and μOR shifts toward TM7, while the displacement of TM6 for M2R and μOR is even smaller than that of the α_2_AR (fig. S6D).

In contrast to the relatively dispersive and sparse polar interactions between α_2_ARs and GoA, hydrophilic residues R380^G.H5.12^, D381^G.H5.13^, and Q384 ^G.H5.16^ on one side of the α5 helix of the Gαs protein form a cluster of hydrogen-bonding interactions with hydrophilic residues from TM3 and TM5 of β_2_AR (Fig. 6, G and H). Notably, because of the shift of the α5 helix of Gαo, its interaction with the TM5 of α_2_ARs is much weaker than that of the β_2_AR-Gs complex (Fig. 6, C to F). Therefore, the TM5 intracellular domain may be essential for the coupling of Gs but not for Gi/o. This observation is consistent with previous functional studies, showing that deletion of residues of the TM5 intracellular end of α_2A_AR entirely ablated the activation of Gs while the activity of Gi was retained (*38*). To further investigate the role of TM5 in the coupling specificity to Gi/o and Gs, we made chimeric constructs by swapping the intracellular domain of TM5 (positions 5.60 to 5.71) between α_2A_AR and β_2_AR. However, we did not observe a switch of coupling selectivity for the two chimeric receptors α_2A_AR- β_2_-TM5 and β_2_AR-α_2A_-TM5 (fig. S7). Although α_2A_AR- β_2_-TM5 showed decreased Gi coupling activity (~3-fold), the Gs activity also decreased substantially (~10-fold) compared with that of the wild-type α_2A_AR (fig. S7, B and C). By contrast, β_2_AR-α_2A_-TM5 showed markedly increased activity for both Gi and Gs coupling (over 100-fold) compared with that of the wild-type β_2_AR (fig. S7, B and C). These data suggest that the TM5 intracellular domain plays important roles in G protein coupling and that the amino acid sequence of α_2A_AR has stronger ability to couple with both G proteins than that of β_2_AR, despite the weaker interactions with G protein (Fig. 6, C to F). It is possible that there are intermediate conformational states of the α_2A_AR-GoA signaling complex where the TM5 forms strong interactions with the Gαo. Such an intermediate state has been observed in other GPCR–G protein signaling complex (*39*). Moreover, these data also suggest that the TM5 intracellular domain is not sufficient to determine the coupling specificity of α_2A_AR and β_2_AR. It is likely that TM5, TM6, and other regions may act simultaneously to determine the G protein coupling specificity and that a possible intermediate state may also play important roles in coupling specificity. It is worthy of note that these functional studies were performed with chimeric Gqi/s proteins and thus may not be able to fully recapitulate the natural behavior of Gi or Gs.

In addition to TM5 and TM6, another major difference between the α_2_ARs-GoA and β_2_AR-Gs structures lies in the interaction between ICL2 and the hydrophobic patch formed by the α5 and αN helices of the G protein. The small residue L/I^34.51^ in α_2_ARs is involved in weak hydrophobic interactions with L195^G.S3.01^, F336^G.H5.08^, and I343^G.H5.15^ from GoA, while in the case of β_2_AR, the bulkier F^34.51^ forms stronger interactions with Gs (Fig. 6, B, F, and H). These observations are similar to several other GPCR-Gi/o complex structures, suggesting that ICL2 may be less important for the coupling with Gi/o but is essential for Gs activation (*29*, *30*, *40*). Indeed, previous mutagenesis studies showed that F^34.51^A mutation can abolish the activity of β_2_AR to activate Gs, while this mutation has little effect on its Gi activity (*41*). Recent functional studies also showed that the I^34.51^A mutation of α_2A_AR can maintain the activation of Gi while abolishing Gs signaling (*9*).

## DISCUSSION

Adrenoceptors are model systems for studying GPCR signaling and important drug targets for a wide variety of diseases. In more recent years, agonists of α_2_ARs have been found to have broad applications in the field of anesthesia and pain management because of their reduced propensity to induce respiratory depression. In the present study, we reported four cryo-EM structures of α_2A_AR signaling complexes bound to the endogenous agonist Norepi and three widely used imidazoline drugs. These structures, in combination with mutagenesis and functional studies, reveal different molecular determinants that recognize Norepi and imidazoline agonists, and key residues including Y431^7.43^, S215^5.42^, and particularly Y409^6.55^, that may lead to a signaling bias of OXY. By comparison with the inactive α_2A_AR, these structures also reveal a key aromatic switch involving F427^7.39^ that plays essential roles in receptor activation, which is consistent with recent structural and biochemical studies (*9*). Comparison with β adrenoceptors shows that the α_2A_AR shares a conserved mechanism for the allosteric coupling between the extracellular ligand binding pocket and the intracellular G protein binding site. However, the activation-associated structural changes in the orthosteric pocket differ between α_2A_AR and β adrenoceptors. There are also substantial differences in the molecular mechanism of Norepi recognition by α_2_ and β adrenoceptors because of several nonconserved residues such as F^7.39^ and Y^6.55^. These signaling complex structures also reveal key regions, including TM5, TM6, and ICL2, that may act together to determine the functional selectivity for Gi/o and Gs for α_2_ARs and β_2_AR, respectively. Nevertheless, the molecular mechanisms of signaling promiscuity at Gi/o and Gs for α_2_AR and β_2_AR are not well elucidated by the current structures. Further structural and biophysical studies on α_2_ARs-Gs and β_2_AR-Gi/o complexes are needed to better understand this dual G protein coupling effect of adrenoceptors. Together, our studies provide a structural framework for understanding the signal transduction of the adrenergic system and are expected to facilitate structure-based drug discovery targeting α_2_ARs.

## MATERIALS AND METHODS

### Expression and purification of α_2A_AR

The human wild-type α_2A_AR was cloned to pFastBac vector with an N-terminal FLAG tag and a C-terminal histidine tag to express in Sf9 insect cells. Recombinant baculovirus for insect cell expression was made using the Bac-to-Bac system. Sf9 cells were grown in SIM SF Medium (Sino Biological Inc.) and were infected with recombinant baculovirus containing the α_2A_AR gene at a density of 4 × 10^6^ cells ml^−1^ in the presence of 5 μM rauwolscine. After 48 hours of infection at 27°C, the cells were spun down and cell pellets were stored at −80°C until use.

Thawed cell pellets were resuspended in hypotonic lysis buffer [10 mM tris, 1 mM EDTA, 5 μM rauwolscine, leupeptin (2.5 μg ml^−1^), and benzamidine (160 μg ml^−1^)]. Cell membranes were then spun down and solubilized with a buffer consisting of 20 mM Hepes (pH 7.5), 500 mM NaCl, 1% n-Dodecyl-b-D-Maltopyranoside (DDM), 0.2% sodium cholate, 0.03% Cholesteryl Hemisuccinate (CHS), 5 μM rauwolscine, leupeptin (2.5 μg ml^−1^), and benzamidine (160 μg ml^−1^). Nickel-NTA sepharose was added to the solubilized receptor and rotated for 2 hours at 4°C. The resin was spun down and washed in batch for three times with a buffer composed of 20 mM Hepes (pH 7.5), 500 mM NaCl, 0.1% DDM, 0.02% sodium cholate, 0.03% CHS, 5 μM rauwolscine, leupeptin (2.5 μg ml^−1^), and benzamidine (160 μg ml^−1^). The washed resin was poured into a glass column, and the receptor was eluted in the wash buffer supplemented with 250 mM imidazole.

The Ni-NTA chromatography–purified receptor was immobilized using anti-flag M1 affinity resin and was extensively washed with a buffer containing 20 mM Hepes (pH 7.5), 500 mM NaCl, 0.1% DDM, 0.02% sodium cholate, 0.003% CHS, 2 mM CaCl_2_, and supplemented with 100 μM agonist. The receptor was subsequently eluted with the same buffer supplemented with flag peptide (0.2 mg ml^−1^) and 5 mM EDTA. The eluted receptor (2 to 3 ml) was concentrated to 500 μl using a 50-kDa molecular weight cutoff Millipore concentrator and Sorvall Legend Micro 17 Microcentrifuge (Thermo Fisher Scientific). The concentrated protein was then loaded on a Superdex 200 size exclusion column (GE Healthcare) with a buffer containing 20 mM Hepes (pH 7.5), 500 mM NaCl, 0.1% DDM, 0.02% sodium cholate, 0.002% CHS, and 100 μM agonist. The monomeric peak fractions of the receptor were collected and concentrated to ~20 mg ml^−1^ and stored at −80°C until use.

### Expression and purification of GoA and scFv16

The GoA protein, which represents the heterotrimeric complex of the guanine nucleotide–binding protein Go subunit α (Gαo), Gβ, and Gγ subunits, was expressed and purified as below. Human Gαo and Gβ1γ2 were synthesized in General Biology company (Hefei). Gαo was cloned into pFastBac vector, Gβ1 with 3C protease-cleavable 6xHis-tag, and Gγ2 was cloned into pFastBac_Dual vector. The GoA was expressed in HighFive insect cells grown in SIM HF Medium (Sino Biological Inc.). Cells were grown to a density of 3 million per milliliter and infected with Gαo and Gβ1γ2 baculovirus at a ratio of 10 to 20 ml liter^−1^ and 1 to 2 ml liter^−1^, respectively. After 48 hours of incubation, the infected cells were harvested by centrifugation and stored at −80°C until use.

Thawed cell pellets were resuspended in lysis buffer [10 mM tris (pH 7.5), 0.1 mM MgCl_2_, 5 mM β-mercaptoethanol (β-ME), 10 μM guanosine diphosphate (GDP), leupeptin (2.5 mg ml^−1^), and benzamidine (160 mg ml^−1^)] and stirred at room temperature for 15 min. Cell membranes were spun down and resuspended with solubilization buffer [20 mM Hepes (pH 7.5), 100 mM NaCl, 1% sodium cholate, 0.05% DDM, 5 mM MgCl_2_, 2 ml CIP, 5 mM β-ME, 15 mM imidazole, 10 μM GDP, leupeptin (2.5 mg ml^−1^), and benzamidine (160 mg ml^−1^)] using a Dounce homogenizer. The samples were stirred at 4°C for 40 min and then centrifuged for 30 min to remove insoluble debris.

Ni-NTA resin preequilibrated in solubilization buffer was added to the supernatant and shaken for 2 hours at 4°C. After incubation, the Ni-NTA resin was spun down and poured into a glass column and washed with 50 ml of solubilization buffer. The heterotrimeric GoA was gradually exchanged into E2 buffer [20 mM Hepes (pH 7.5), 50 mM NaCl, 0.1% DDM, 1 mM MgCl_2_, 5 mM β-ME, 10 μM GDP, leupeptin (2.5 mg ml^−1^), and benzamidine (160 mg ml^−1^)]. The protein was then eluted with E2 buffer supplemented with 250 mM imidazole.

The sample was then dephosphorylated by treating with 5 μl lambda phosphatase (supplemented with 1 mM MnCl_2_ for activity; New England Biolabs) and incubated at 4°C for 30 min. The Ni-NTA chromatography–purified GoA was further purified with a MonoQ column (GE Healthcare). The peak fractions of the MonoQ chromatography were collected and exchanged to E3 buffer [20 mM Hepes (pH 7.5), 100 mM NaCl, 0.1% DDM, 1 mM MgCl_2_, 10 μM GDP, and 50 μM Tirs(2-carboxyethy)phosphine (TCEP)] by repeated concentration and dilution using a 50-kDa molecular weight cutoff Millipore concentrator. The concentrated heterotrimeric GoA was aliquoted, flash frozen in liquid nitrogen, and stored at −80°C before use.

The scFv16 gene was cloned into pFastBac vector with a C-terminal histidine tag and expressed in secreted form in HighFive insect cells using the Bac-to-Bac system (Thermo Fisher Scientific). The protein was purified with Ni-NTA column followed by size exclusion chromatography using a Superdex 200 Increase 10/300GL column (GE Healthcare). The monomeric peak fractions were collected, concentrated, and stored at −80°C until use.

### α_2A_AR–GoA complex formation

The complex of α_2A_AR with heterotrimeric GoA was formed in a buffer composed of 20 mM Hepes (pH 7.5), 100 mM NaCl, 0.1% DDM, 1 mM MgCl_2_, 10 μM GDP, and 100 μM agonist. The α_2A_AR-GoA complex was then treated with 50 U of apyrase (NEB) on ice overnight and exchanged on an anti-Flag M1 column into a buffer containing 20 mM Hepes (pH 7.5), 100 mM NaCl, 0.0075% lauryl maltose neopentyl glycol (MNG, NG310 Anatrace), 0.0025% GDN (GDN101, Anatrace), 0.001% CHS, 100 μM agonist, and 2 mM CaCl_2_ in a stepwise manner. After elution by adding 5 mM EDTA and Flag peptide (0.2 mg ml^−1^), the complex was concentrated and incubated with excess scFv16 for 1 hour on ice and then loaded onto Superdex 200 Increase 10/300GL column (GE Healthcare) with a running buffer of 20 mM Hepes (pH 7.5), 100 mM NaCl, 0.00075% MNG, 0.00025% GDN, 0.0001% CHS, and 100 μM agonist. The monomeric peak fraction of the α_2A_AR-GoA complex was collected and concentrated to ~10 mg/ml for cryo-EM.

### Cryo-EM sample preparation and data collection

The gold film (*42*) (UltraAuFoil, 300 mesh, R1.2/1.3) or amorphous alloy film (*43*) (300 mesh, R1.2/1.3, Zhenjiang Lehua Electronic Technology Co. Ltd.) was glow discharged with air for 40 s at 15 mA at easiGlow Glow Discharge Cleaning System (PELCO, USA). Three microliters of purified complex sample was dropped onto the grid and then blotted for 3.5 s with blotting force 0 and plunged into liquid ethane cooled by liquid nitrogen using Vitrobot Mark IV (Thermo Fisher Scientific, USA). Cryo-EM datasets were collected with the 300-kV Titan Krios Gi3 microscope. The raw movies were collected by Gatan K3 BioQuantum Camera at magnification of 105,000, with a pixel size of 0.85 Å. Inelastically scattered electrons were excluded by a GIF Quantum energy filter (Gatan, USA) using a slit width of 20 eV. The movies were acquired with the defocus range of −1.0 to −2.0 μm with total exposure time of 2.5 s fragmented into 50 frames and with the dose rate from 17.36 to 17.65 e per pixel per second. SerialEM (*44*) was used for semiautomatic data acquisition.

### Cryo-EM data processing, model building, and refinement

The image stacks were collected and subjected for motion correction using MotionCor2 (*45*). Contrast transfer function parameters were estimated by CTFFIND4 (*46*), implemented in RELION3.1 (*47*). Particles were auto-picked from micrographs by RELION and then subjected to two-dimensional (2D) classification using cryoSPARC (*48*). Selected particles with an appropriate 2D average from 2D classification were further subjected to 3D classification using RELION with an initial model of the α_2B_AR-GoA-scFv16 complex (*27*). Eventually, particles with high-resolution 3D average were selected from 3D classification, which result in a map with initial resolution of near atomic level by 3D auto-refinement. The refined particles were subjected to CTF refinement to update per-particle defocus and per-micrograph astigmatism. Bayesian polishing, which means local motion correction, was then performed on the refined particles. Another round of CTF refinement was performed on the shiny particles generated from polishing followed by another round of 3D auto-refinement and, lastly, yielded a series of final map with resolution from 3.0 to 3.6 Å after postprocessing determined by gold standard Fourier shell correlation using the 0.143 criterion. The local resolution map was calculated from the Bsoft package (*49*) using two unfiltered half maps.

### Functional assays

The human wild-type α_2A_AR, β_2_AR, and respective receptor mutants or chimeras all carrying an N-terminal HA-signal sequence and a FLAG-tag (*50*) were cloned to pCDNA3.1 for G protein activation assays or fused to the ARMS2-PK2 sequence and cloned to pCMV (DiscoverX, Eurofins) for β-arrestin-2 recruitment assays, respectively, using polymerase chain reaction and Gibson Assembly (New England Biolabs) (*51*). Sequence integrity was verified by DNA sequencing (Eurofins Genomics).

The determination of receptor-mediated G protein signaling by wild-type and mutant receptors was performed applying an IP accumulation assay (IP-One HTRF, Cisbio, Codolet, France) according to the manufacturer’s protocol and in analogy to previously described protocols (*52*, *53*). In brief, human embryonic kidney (HEK) 293T cells were cotransfected with the cDNA coding for α_2A_AR, α_2A_AR-D128^3.32^A, α_2A_AR-S215^5.42^A, α_2A_AR-Y409^6.55^A, α_2A_AR-F427^7.39^A, or α_2A_AR-Y431^7.43^A, α_2A_AR-β_2_-TM5, or β_2_AR-α_2A_-TM5, respectively, and the hybrid G protein Gαqi or Gαqs (Gαq protein with the last five amino acids at the C terminus replaced by the corresponding sequence of Gαi or Gαs, respectively; gift from The J. David Gladstone Institutes, San Francisco, CA) in a ratio of 1:2 and transferred into 384-well microplates. On the day of the experiment, incubation started by adding the agonists for 90 min (α_2A_AR, α_2A_AR-S215^5.42^A, and α_2A_AR-F427^7.39^A) or 150 min (α_2A_AR-D128^3.32^A, α_2A_AR-Y409^6.55^A, and α_2A_AR-Y431^7.43^A), or 120 min (for the direct comparison of α_2A_AR and β_2_AR with the α_2A_AR-β_2_-TM5 and β_2_AR-α_2A_-TM5 chimeras), respectively. Accumulation of second messenger was stopped by addition of the detection reagents (IP1-d2 conjugate and anti-IP1cryptate TB conjugate). After a further 60 min, time-resolved fluorescence resonance energy transfer (FRET) was measured using the Clariostar plate reader (BMG, Ortenberg, Germany). FRET signals were calculated as the ratio of emissions at 665 and 620 nm and ligand-induced changes in FRET (deltaFRET) normalized to the maximum effect of norepinephrine (100%) and vehicle (0%). For the mutant α_2A_AR-D128^3.32^A, the maximum effect of dexmedetomidine was used as 100%. Normalized concentration-response curves from 4 to 11 experiments each done in duplicates were analyzed using the algorithms for four-parameter nonlinear regression implemented in PRISM 8.0 (GraphPad Software, USA) to derive EC_50_ and *E*_max_ values.

Determination of receptor stimulated β-arrestin-2 recruitment was performed applying the PathHunter assay (DiscoverX, Birmingham, UK) measuring fragment complementation of β-galactosidase as described (*53*, *54*). In detail, HEK293T cells were cotransfected with α_2A_AR wild-type or mutant receptor each fused to the ARMS2-PK2 fragment for enzyme complementation and GRK2 in a ratio of 1:1 and transferred into 384-well microplates. Measurement was started by incubating the cells with the agonists for 60 min (α_2A_AR, α_2A_AR-S215^5.42^A, α_2A_AR-Y409^6.55^A, α_2A_AR-F427^7.39^A, or α_2A_AR-Y431^7.43^A) or 150 min (α_2A_AR-D128^3.32^A), respectively. Chemoluminescence was monitored with a Clariostar plate reader and analyzed by normalizing the raw data relative to basal activity (0%) and the maximum effect of norepinephrine (100%). For the mutant α_2A_AR-D128^3.32^A, normalization was done by analyzing the raw data as fold change relative to basal (=100%). Three to nine individual experiments each done in duplicate were analyzed by nonlinear regression, applying the algorithms in Prism 8.0 (GraphPad, San Diego, CA) to get dose-response curves representing average EC_50_ and *E*_max_ values.

Receptor surface expression was analyzed using an enzyme-linked immunosorbent assay directed against the N-terminal FLAG-tag as previously described (*55*). In brief, HEK293T cells were diluted to a density of 2 × 10^5^ cells ml^−1^ in Dulbecco’s modified Eagle’s medium/F12 supplemented with 10% fetal bovine serum and transfected with wild-type α_2A_AR, β_2_AR, or respective receptor mutants in pcDNA3.1 or pCMV using polyethyleneimine (PEI) at a 3:1 PEI/DNA ratio. Per 1.2 ml of cell suspension, 200 ng of α_2A_AR and 800 ng of single-stranded salmon sperm DNA (Sigma-Aldrich) were used. Fifty thousand cells per well were transferred to a 48-well plate coated with poly-d-lysine and incubated at 37°C and 5% CO_2_ for 48 hours. Cells were fixated using 4% paraformaldehyde (200 μl, 10 min, room temperature), washed once (wash buffer, 300 μl, 150 mM NaCl, 25 mM tris, pH 7.5), and blocked for 60 min [skim milk powder (30 g liter^−1^) in wash buffer], before anti-FLAG mouse immunoglobulin G (IgG; Sigma-Aldrich; 1:4000 in blocking solution, 200 μl) was added. After 60 min, wells were washed twice (300 μl) and blocked for a further 60 min, before 200 μl of anti-mouse rabbit IgG-HPR (Sigma-Aldrich; 1:20,000 in blocking solution) was added. After 60 min, cells were washed thrice, before the addition of substrate buffer (300 μl, 2.8 mM *o*-phenylenediamine in 35 mM citric acid, 66 mM Na_2_HPO_4_, pH 5.0). Reactions were kept in the dark for 5 to 10 min and stopped by addition of 1 M H_2_SO_4_ (200 μl). The resulting solution (150 μl) was transferred to clear, flat bottom 96-well plates, and absorption at 492 nm was measured with the Clariostar microplate reader. Data were normalized to the expression level of wild-type α_2A_AR (100%) and mock transfected HEK293T cells (0%), respectively. *N* = 5 independent experiments were performed, with each condition in triplicate.

## Notes

View/request a protocol for this paper from *Bio-protocol*.
