# Supplementary material for: Structural insights into ligand recognition, activation, and signaling of the α2A adrenergic receptor
Source: Sci Adv. 2022 Mar 4;8(9):eabj5347. doi: 10.1126/sciadv.abj5347 (PMC8896805; doi:10.1126/sciadv.abj5347)
Supplement: Supplementary file 1 — Figs. S1 to S7 Tables S1 and S2 [file sciadv.abj5347_sm.pdf]

Science Advances
AAAS

Supplementary Materials for

Structural insights into ligand recognition, activation, and signaling of the α_{2A} adrenergic receptor

Jun Xu, Sheng Cao, Harald Hübner, Dorothée Weikert, Geng Chen, Qiuyuan Lu,
Daopeng Yuan*, Peter Gmeiner*, Zheng Liu*, Yang Du*

*Corresponding author. Email: daopengyuan@126.com (D.Y.); peter.gmeiner@fau.de (P.G.);
liuzheng@cuhk.edu.cn (Z.L.); yangdu@cuhk.edu.cn (Y.D.)

Published 4 March 2022, *Sci. Adv.* **8**, eabj5347 (2022)
DOI: 10.1126/sciadv.abj5347

This PDF file includes:

Figs. S1 to S7
Tables S1 and S2

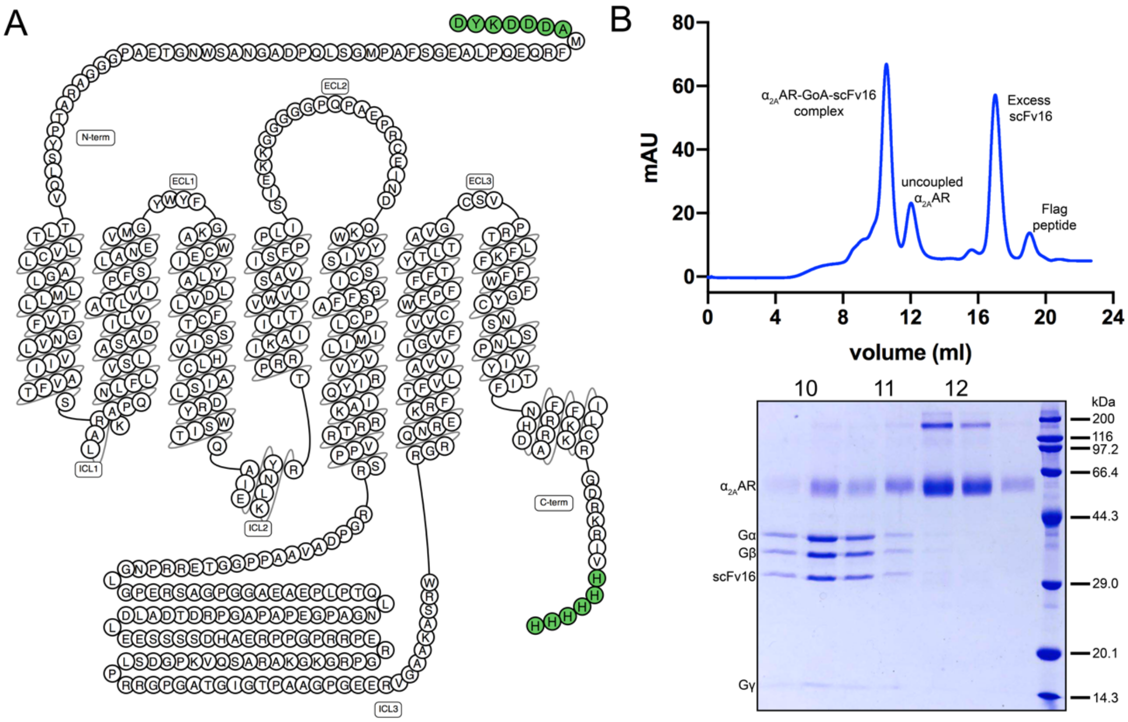

Fig. S1. Construct and complex formation. (A) Snake plot of secondary structure and amino acid sequence of the α_{2A}AR. The N-terminal flag-tag and C-terminal histidine tag are highlighted in green. **(B)** Representative size exclusion chromatography profile and SDS-PAGE of agonist-bound α_{2A}AR-GoA-scFv16 complex.

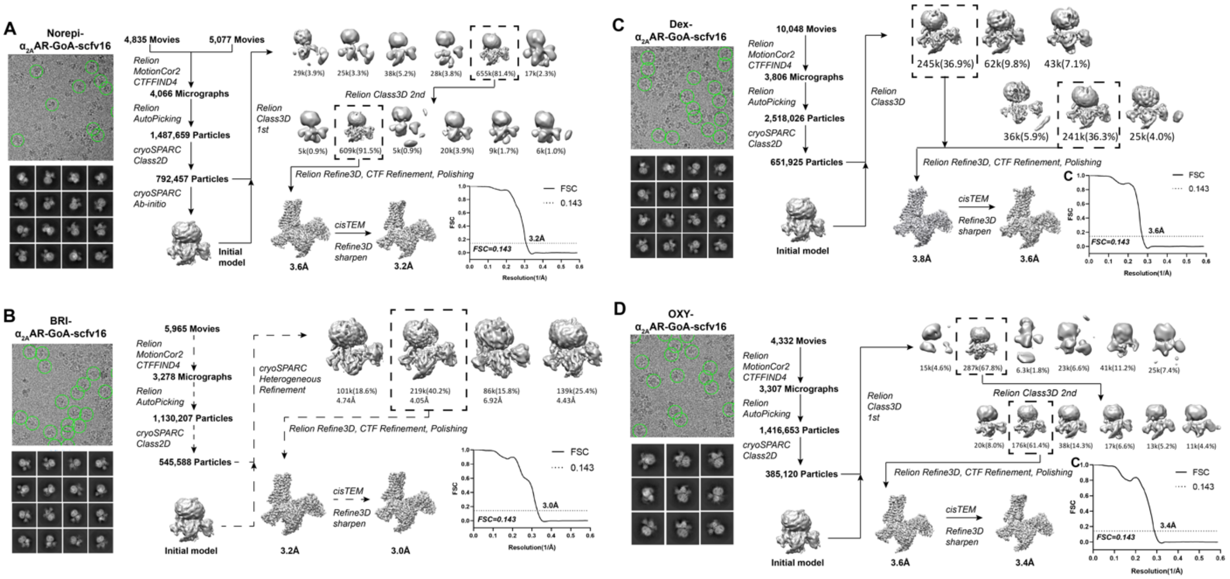

Fig. S2. Cryo-EM data processing. Representative cryo-EM image, representative 2D classifications displaying distinct structural features from different views and work-flow of cryo-EM data processing for α_{2A}AR-GoA-scFv16 complex bound to Norepi **(A)**, BRI **(B)**, DEX **(C)** and OXY **(D)**, respectively. 'Gold standard' FSC curve indicates overall nominal resolution at 3.2 Å, 3.0 Å, 3.6 Å and 3.4Å using the FSC = 0.143 criterion.

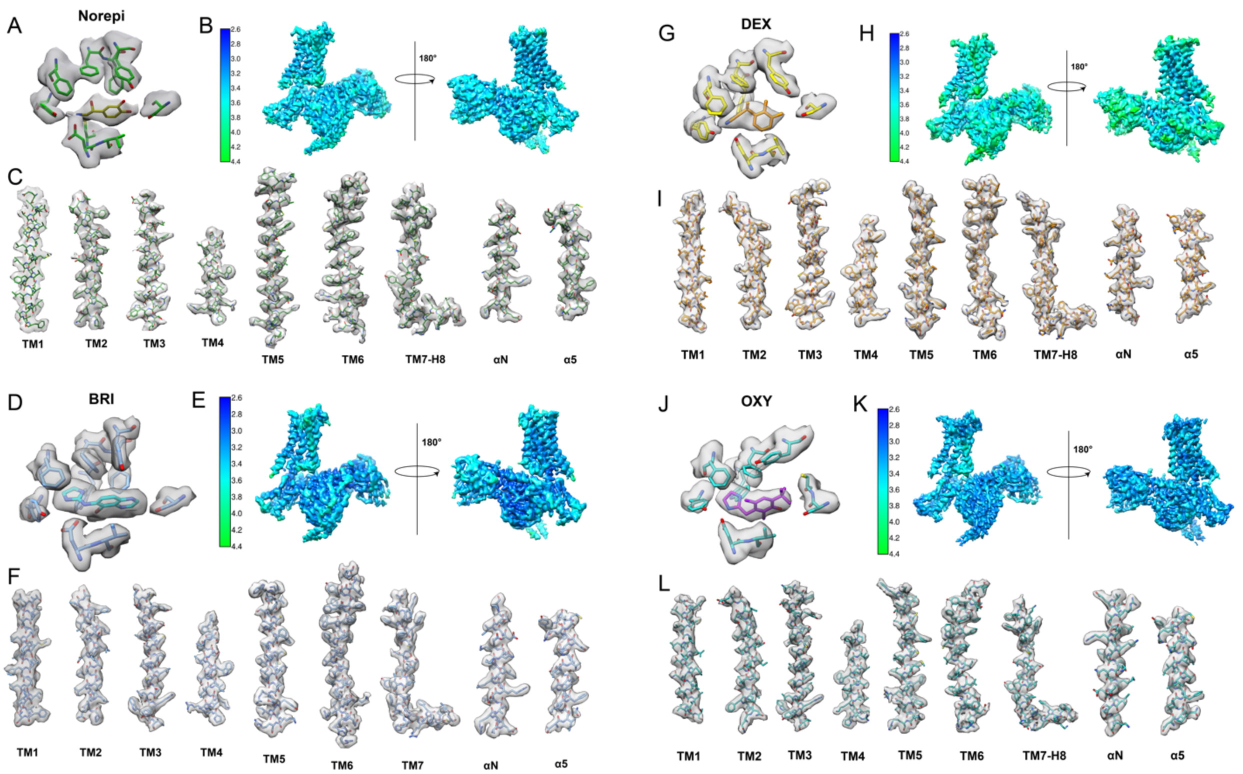

Fig. S3. Cryo-EM maps and refined structures. Density maps for residues in ligand binding pocket, local resolution maps and the model of all transmembrane helices and helix 8 of α_{2A}AR as well as α5 and αN helices of Gαo for α_{2A}AR-GoA-scFv16 complex bound to Norepi **(A-C)**, BRI **(D-F)**, DEX **(G-I)** and OXY **(J-L)**, respectively.

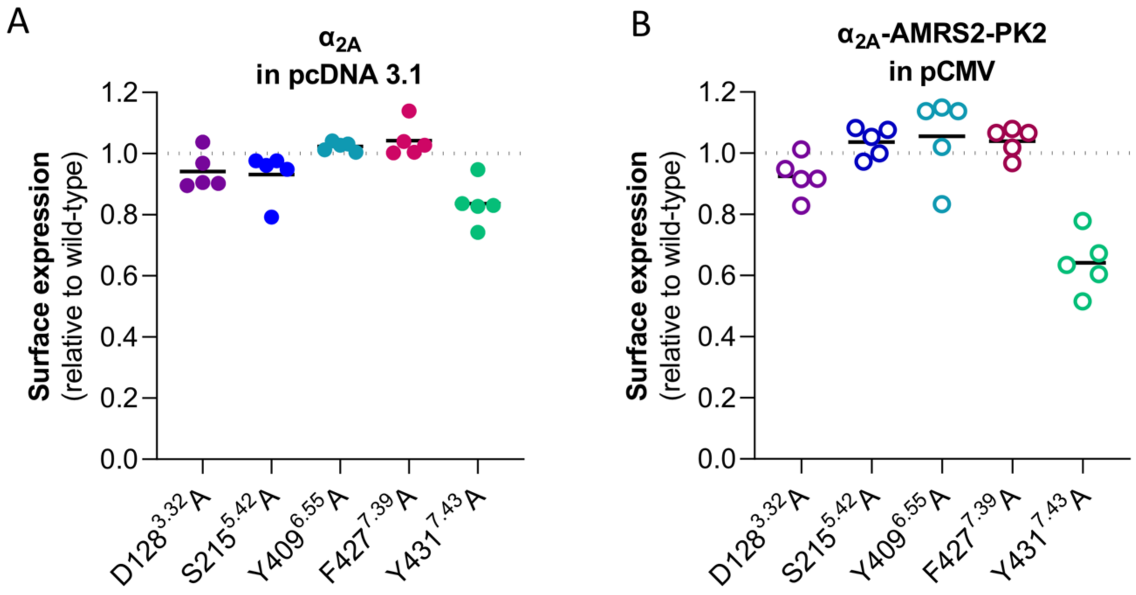

Fig. S4. Surface expression of α_{2A}AR mutants. The influence of point mutations on receptor surface expression in HEK293T cells was assessed by ELISA directed against the N-terminal FLAG-tag. Point mutations D128$^{3.32}$A, S215$^{5.42}$A, Y409$^{6.55}$A, and F427$^{7.39}$A have no substantial influence on the surface expression of α_{2A}AR cloned into pcDNA3.1 (**A**, closed circles) or the ARMS2-PK2-tagged receptor variant (**B**, open circles) used in β-arrestin-2 recruitment assays. Mutation Y431$^{7.43}$A decreases surface expression of α_{2A}AR receptors to 84% and 64% relative to wild type, respectively. The graphs show the individual data points and the mean of n = 5 independent experiments, each performed in triplicates.

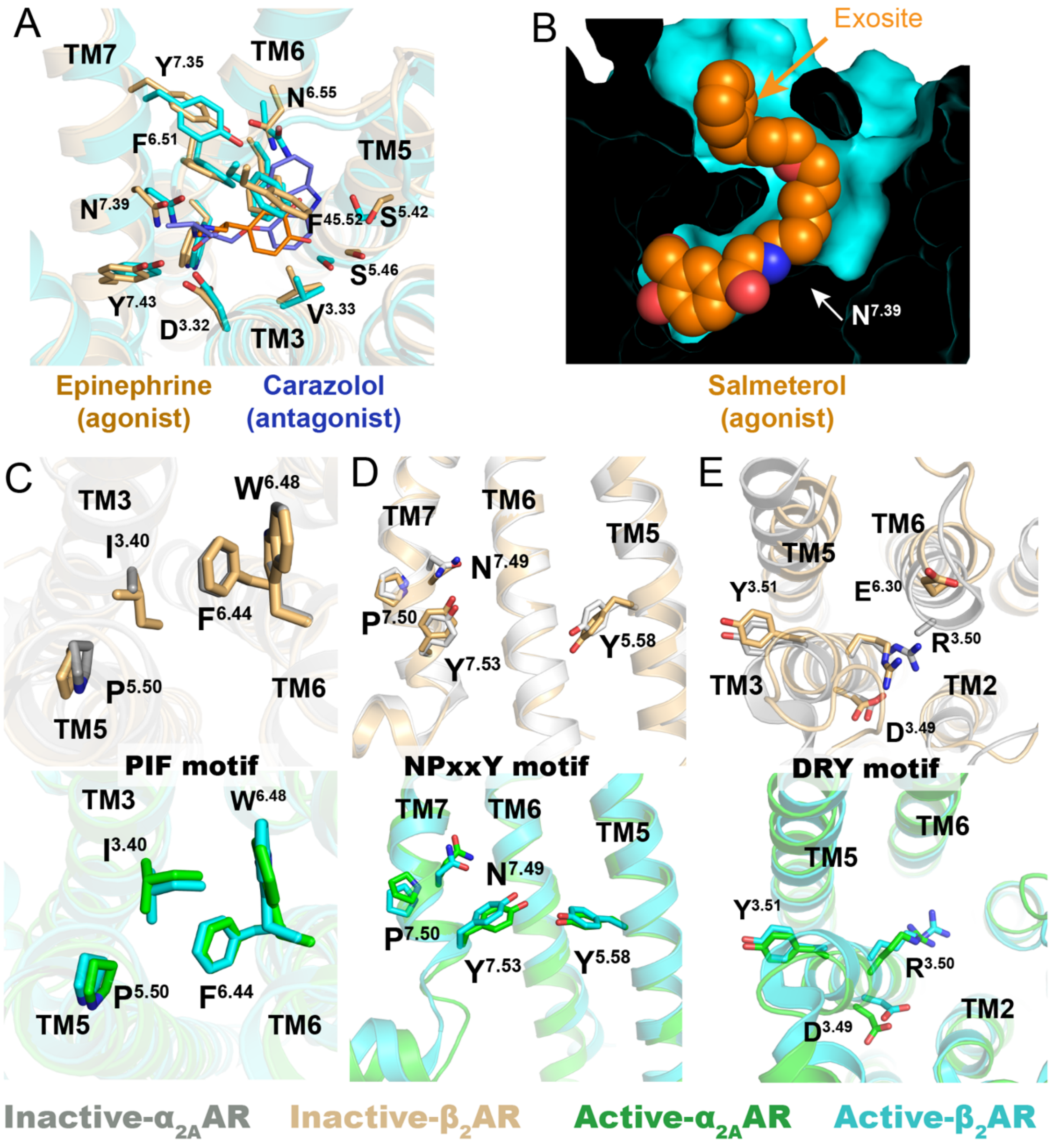

Fig. S5. Orthosteric pocket of β_2AR and conformational changes of conserved micro-switches. (A) Comparison of the orthosteric pocket of β_2AR bound to the antagonist carazolol and the agonist epinephrine. **(B)** Cross-sections of β_2AR bound to the agonist salmeterol are shown, with the interior in black and the exosite highlighted. **(C-E)** Comparison of the P-I-F motif **(C)**, NPxxY motif **(D)** and DRY motif **(E)** for α_{2A}AR and β_2AR in inactive and active states.

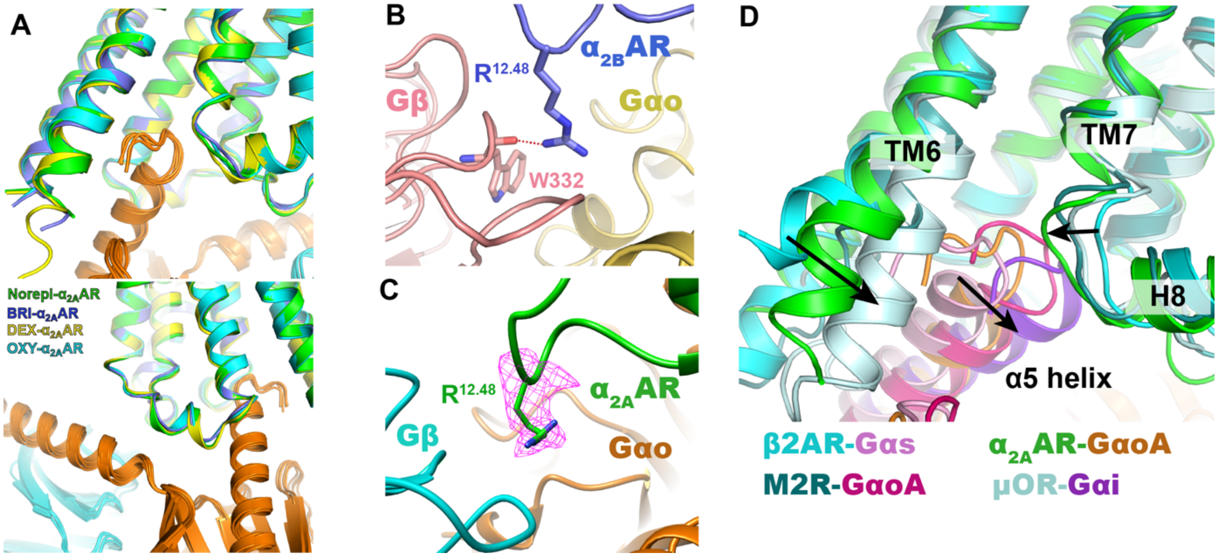

Fig. S6. Comparison of G-protein binding interface. (A) Comparison of the interfaces of the α_{2A}AR-GoA complexes bound to four different drugs. **(B)** Polar interaction between ICL1 of α_{2B}AR and Gβ. **(C)** Interface between ICL1 of α_{2A}AR with Gβ, density of $R^{12.48}$ is depicted as magenta meshes. **(D)** Comparison of the coupling interfaces of G protein α5 helix with different receptors.

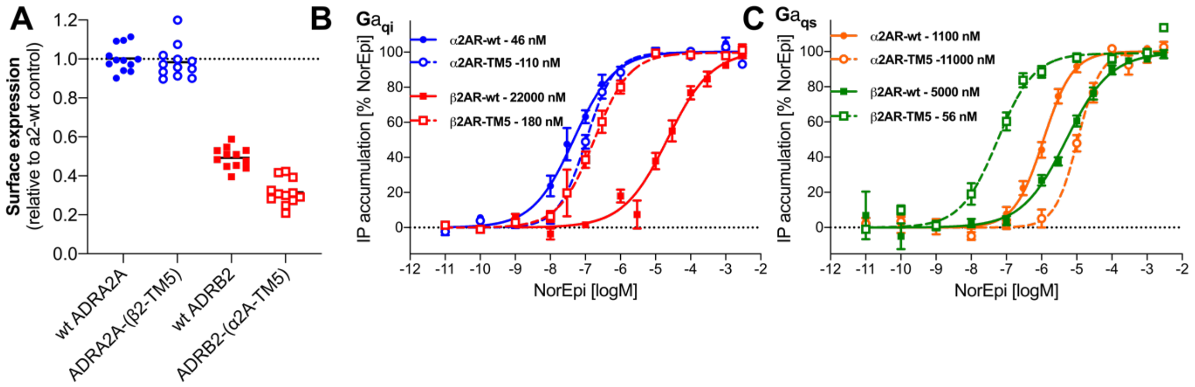

Fig. S7. Functional analysis of α_{2A}AR-β_2AR TM5 chimera. (A) The influence of chimeric mutations on receptor surface expression in HEK293T cells was assessed by ELISA directed against the N-terminal FLAG-tag, mean and individual data points. (B) Concentration-response curves of chimera and wild type α_{2A}AR and β_2AR toward Gqi protein activation. (C) Concentration-response curves of chimera and wild type α_{2A}AR and β_2AR toward Gqs protein activation. Data are presented as mean ± SEM of nine to fourteen independent experiments with repeats in duplicate.

Table S1. Cryo-EM data collection, refinement and validation statistics

**Data collection and processing**	**Norepi-α_{2A}AR-GoA-scfv16 EMBD-31147, PDB ID 7EJ0**	**BRI-α_{2A}AR-GoA-scfv16 EMBD-31156, PDB ID 7EJ8**	**DEX-α_{2A}AR-GoA-scfv16 EMBD-31157, PDB ID 7EJA**	**OXY-α_{2A}AR-GoA-scfv16 EMBD-31162, PDB ID 7EJK**
Magnification	105,000	105,000	105,000	105,000
Voltage (kV)	300	300	300	300
Electron exposure (e^-/Å)	17.65	17.53	17.46	17.36
Defocus range (μm)	-1.0~-2.0	-0.8~-1.8	-1.0~-2.0	-0.8~-1.8
Pixel size (Å)	0.85	0.85	0.85	0.85
Symmetry imposed	C1	C1	C1	C1
Initial particle images (no.)	1487659	1130207	2518026	1416653
Final particle images (no.)	261073	219173	455506	220513
Map resolution (Å)	3.2	3.0	3.6	3.4
FSC threshold	0.143	0.143	0.143	0.143
**Refinement**				
Initial model used (PDB code)	6K41	6K41	6K41	6K41
Map sharpening B-factor (Å)	-100	-60	-130	-90
**Model composition**				
Non-hydrogen atoms	8600	8647	8584	8586
Protein residues	1099	1100	1097	1093
**B factor (Å)**				
Protein	131.13	120.81	180.56	110.01
Ligand	122.43	135.93	141.72	117.36
**R.m.s. deviations**				
Bond lengths (Å)	0.009	0.007	0.004	0.005
Bond angles (°)	1.103	0.845	0.841	1.068
**Validation**				
MolProbity score	2.23	2.09	2.14	1.95
Clashscore	20.58	15.57	17	11.94
**Ramachandran plot**				
Favored (%)	93.51	94.17	93.95	94.78
Allowed (%)	6.49	5.83	6.05	5.22
Disallowed (%)	0	0	0	0

Table S2 Activation data for norepinephrine Noepi, BRI, DEX and OXY in G protein signaling and arrestin recruitment at α_{2A}AR wild-type receptor and the mutants D128$^{3.32}$A, S215$^{5.42}$A, Y409$^{6.55}$A, F427$^{7.39}$A and Y431$^{7.43}$A, respectively.

receptor	assay	Norepi			BRI			DEX			OXY		
		EC_{50} [nM][a]	E_{max} [%][b]	n[c]	EC_{50} [nM][a]	E_{max} [%][b]	n[c]	EC_{50} [nM][a]	E_{max} [%][b]	n[c]	EC_{50} [nM][a]	E_{max} [%][b]	n[c]
wt	$G\alpha_{qi}$	17 ± 4	100	10	0.30 ± 0.09	93 ± 2	6	0.33 ± 0.06	99 ± 2	11	0.63 ± 0.11	89 ± 3	6
	β-arrestin	98 ± 20	100	9	12 ± 3.4	104 ± 3	4	3.9 ± 0.6	92 ± 2	9	5.9 ± 1.6	55 ± 4	5
D128$^{3.32}$A	$G\alpha_{qi}$	-	17 (1 mM)[d,e]	6	-	10 (300 µM)[d,e]	5	30000 ± 4900	100[d]	6	52000 ± 8100	98 ± 4[d]	6
	β-arrestin	-	<1.1-fold[f]	4	-	<1.1-fold[f]	3	-	<1.1-fold[f]	4	-	<1.1-fold[f]	4
S215$^{5.42}$A	$G\alpha_{qi}$	560 ± 150	100	5	0.57 ± 0.13	92 ± 3	5	0.22 ± 0.07	92 ± 4	4	0.099 ± 0.03	99 ± 4	5
	β-arrestin	30000 ± 4700	100	7	21 ± 4.0	102 ± 4	6	2.4 ± 0.58	99 ± 4	7	5.0 ± 3.6	84 ± 2	6
Y409$^{6.55}$A	$G\alpha_{qi}$	6400 ± 680	100	6	250 ± 54	85 ± 5	6	2.3 ± 0.34	101 ± 2	6	0.26 ± 0.05	94 ± 5	6
	β-arrestin	170000 ± 32000	100	7	-	<14 (30 µM)[e]	5	79 ± 9.6	112 ± 4	6	2.6 ± 0.76	84 ± 2	7
F427$^{7.39}$A	$G\alpha_{qi}$	450 ± 65	100	6	680 ± 170	65 ± 2	5	240 ± 70	99 ± 3	5	65 ± 15	78 ± 5	5
	β-arrestin	12000 ± 890	100	6	28000 ± 3000	25 ± 3	6	26000 ± 7200	66 ± 5	5	-	<5	4
Y431$^{7.43}$A	$G\alpha_{qi}$	75000 ± 12000	100	6	560 ± 89	91 ± 5	5	6800 ± 3400	124 ± 17	5	17000 ± 2900	78 ± 3	5
	β-arrestin	280000 ± 69000	100	6	8800 ± 1600	127 ± 9	5	-	<5	4	-	<5	3

[a] Potency displayed as mean EC_{50} value in nM ± SEM. [b] Maximum effect in % ± SEM relative to the effect of the reference agonist norepinephrine. [c] Number of individual experiments each done in duplicates. [d] Normalization of E_{max} was analyzed to the maximum effect of dexmedetomidine (=100%). [e] No complete dose-response curve; maximum efficacy at the indicated concentration (in brackets). [f] No arrestin recruitment detectable for the tested agonists at mutant D128$^{3.32}$A. Normalization was done by referring activity relative to basal value (x-fold).
